# Research Progress in Chinese Herbal Medicines for Treatment of Sepsis: Pharmacological Action, Phytochemistry, and Pharmacokinetics

**DOI:** 10.3390/ijms222011078

**Published:** 2021-10-14

**Authors:** Chen Cheng, Xuan Yu

**Affiliations:** State Key Laboratory of Drug Research, Shanghai Institute of Materia Medica, Chinese Academy of Sciences, 501 Haike Road, Zhangjiang Hi-Tech Park, Shanghai 201203, China; 201728012342061@simm.ac.cn

**Keywords:** antiseptic actions, Chinese herbal medicines, material basis, Qingwen Baidu decoction, Shengfu injection, Shengmai, sepsis, Xuanbai Chengqi decoction, XueBiJing injection

## Abstract

Sepsis is a life-threatening organ dysfunction caused by a dysregulated host response to infection; the pathophysiology of sepsis is complex. The incidence of sepsis is steadily increasing, with worldwide mortality ranging between 30% and 50%. Current treatment approaches mainly rely on the timely and appropriate administration of antimicrobials and supportive therapies, but the search for pharmacotherapies modulating the host response has been unsuccessful. Chinese herbal medicines, i.e., Chinese patent medicines, Chinese herbal prescriptions, and single Chinese herbs, play an important role in the treatment of sepsis through multicomponent, multipathway, and multitargeting abilities and have been officially recommended for the management of COVID-19. Chinese herbal medicines have therapeutic actions promising for the treatment of sepsis; basic scientific research on these medicines is increasing. However, the material bases of most Chinese herbal medicines and their underlying mechanisms of action have not yet been fully elucidated. This review summarizes the current studies of Chinese herbal medicines used for the treatment of sepsis in terms of clinical efficacy and safety, pharmacological activity, phytochemistry, bioactive constituents, mechanisms of action, and pharmacokinetics, to provide an important foundation for clarifying the pathogenesis of sepsis and developing novel antisepsis drugs based on Chinese herbal medicines.

## 1. Introduction

Sepsis is life-threatening organ dysfunction caused by a dysregulated host response to infection [1]. A meta-analysis showed that the 10-year incidence (2005–2015) of sepsis and severe sepsis in developed countries was 437 and 270 per 100,000 population per year, respectively, while mortality was 17% and 26%, respectively [2]. Severe sepsis was defined as a host’s systemic inflammatory response syndrome (SIRS) to infection complicated by organ dysfunction. The terms SIRS and severe sepsis were deleted in February 2016, when the European Society of Intensive Care Medicine and the Society of Critical Care Medicine (SCCM) published new consensus definitions of sepsis and related clinical criteria (Sepsis-3) [1]. Sepsis has exceeded myocardial infarctions in terms of the mortality rate and has become a major cause of death in non-cardiac patients in intensive care units (ICUs). According to a domestic epidemiological investigation, the incidence of sepsis and the 90-day mortality rate among ICU patients in 44 Chinese hospitals were 20.6% and 35.5%, respectively, while the mortality was as high as 50% or above for severe cases [3]. Coronavirus disease 2019 (COVID-19) created a global public health emergency since its original outbreak at the end of 2019 [4]. The ongoing COVID-19 pandemic has infected over 20,000,000 people around the world, claiming the lives of nearly 5 million people worldwide. Among the patients hospitalized with COVID-19, 26% have been treated as critical cases, which could involve sepsis or even septic shock [5]. Septic shock is a subset of sepsis in which underlying circulatory and cellular–metabolic abnormalities are profound enough to substantially increase mortality, and operationally defined as requiring vasopressor therapy to maintain a mean arterial blood pressure of >65 mmHg and an increased plasma lactate level of >2 mmol/L [1,6]. The proposed criteria for sepsis and septic shock were summarized by Hotchkiss et al. and Gotts et al. [6,7].

Due to the high incidence and mortality of sepsis [3], its diagnosis and treatment have been the focuses of critical medicine, emergency medicine, and infectious diseases studies. Sepsis diagnoses (versions 1.0, 2.0, and 3.0), as well as various treatment plans, have been proposed by experts since 1992 [1,8,9]. Sepsis is commonly characterized by complex mechanisms that involve coagulation abnormalities, the uncontrolled release of inflammatory mediators, an excessive innate immune response, endothelial capillary leakage syndrome, organ dysfunction, etc. [10,11]. The current treatment approaches mainly rely on the timely and appropriate administration of antimicrobials, as well as supportive therapies [12]. Although the pathophysiology of sepsis is now much better understood, the search for pharmacotherapies for modulating the septic response has been unsuccessful, and the incidence and mortality of sepsis have not significantly decreased over the past two decades [13,14].

To treat both the symptoms and internal causes of disease, traditional Chinese medicine (TCM) advocates a harmonious, balanced state of the body; it provides advantages in treating different stages of sepsis [15]. Chinese herbal medicines (CHMs) can inhibit platelet aggregation, regulate inflammation and the immune response, and improve microcirculation to thus prevent the progression of sepsis and improve the prognosis of sepsis patients. Such medicines include Chinese patent medicines, Chinese herbal prescriptions, and single Chinese herbs. In the early stage of sepsis, a combination of CHM with antibiotics could reduce the occurrence of drug-resistant bacteria, especially for patients with drug-resistant infections [15]. With the development of sepsis, the occurrence of multiple organ dysfunction syndrome (MODS) can be reduced by strengthening the stomach and spleen through increasing lucidity and decreasing turbidity. If MODS occurs, CHM can also be used to fight against septic shock and even organ dysfunction [16].

Based on the characteristics of emergency medicine in China, the Preventing Sepsis Campaign in China (PSCC) was initiated in May 2018 [17]. It was advocated by experts that the prevention, diagnosis, and treatment of sepsis should be performed as early as possible to decrease morbidity and mortality, and the principle of the prevention of sepsis was introduced to prevent its occurrence. Several Chinese treatment guidelines for sepsis management and expert consensus—e.g., the Chinese guidelines for the emergency management of sepsis and septic shock 2018, the clinical practice guidelines on traditional Chinese medicine therapy alone or combined with antibiotics for sepsis, and the Chinese emergency medicine expert consensus on the diagnosis and treatment of sepsis complicated by disseminated intravascular coagulation—have been successively released for the management of sepsis [16,17,18]. In these treatment guidelines and expert agreements, CHMs are recommended as add-on therapies to complement the conventional treatment of sepsis, e.g., a XueBiJing injection (XBJ) for sepsis, a ShenFu injection (SF) for septic shock, the ShengMai formula (SMF) for sepsis with the qi and yin exhaustion pattern, the Xuanbai Chengqi decoction (XBCQ) for sepsis with acute respiratory distress syndrome (ARDS), the Qingwen Baidu decoction (QWBD) for sepsis with the internal exuberance of toxins and heat pattern, etc. [15]. The diagnosis and treatment protocol for COVID-19 (the revised eighth version) released by China’s National Health Commission also recommends the use of CHM in accordance with different degrees of severity of COVID-19 [19]. XueBiJing, ShenFu, and ShengMai injections are typical herbal injections officially recommended for the management of COVID-19 when patients with a severe case of the disease develop SIRS and/or MODS [19].

Although CHMs have been widely used in the clinic for the treatment of sepsis, their material bases and underlying mechanisms of action have not yet been well defined. Here, we summarize the research progress for several of the most frequently used antisepsis CHM, including their clinical efficacy, pharmacodynamics, mechanisms of action, phytochemistry, and pharmacokinetics. This review provides a theoretical basis for their clinical application and provides important information for further clarifying the material bases of such medicines, aiming to provide potential bioactive and bioavailable compounds for developing novel antisepsis drugs.

## 2. Chinese Herbal Medicines

### 2.1. Chinese Patent Medicines

#### 2.1.1. XueBiJing Injection

The XueBiJing injection (XBJ), derived from the Xuefu Zhuyu decoction, is an injectable licensed in China since 2004 for sepsis and MODS, with a National Medical Products Administration (NMPA) drug ratification number of GuoYaoZhunZi-Z20040033. It is exclusively manufactured by Tianjin Chasesun Pharmaceutical (Tianjin, China). The herbal injection is prepared from a combination of *Carthamus tinctorius* flower (Honghua in Chinese), *Paeonia lactiflora* root (Chishao), *Salvia miltiorrhiza* root (Danshen), *Ligusticum chuanxiong* rhizome (Chuanxiong), and *Angelica sinensis* root (Danggui). Several meta-analyses have suggested that the addition of XBJ to routine sepsis care could further reduce the 28-day mortality of patients and incidence of complications, and improve patient prognosis [20,21,22,23,24]. The 28-day mortality is the primary clinical outcome of sepsis care. In a prospective, randomized controlled trial in 710 patients with severe community-acquired pneumonia, adding XBJ to the conventional treatment reduced the 28-day mortality from 24.6% (conventional treatment) to 15.9% (conventional treatment + XBJ), increased the percentage of patients with improved pneumonia severity indices from 46.3% to 60.8%, and improved their Sequential Organ Failure Assessment (SOFA) scores from 4.44 to 3.65 and Acute Physiology and Chronic Health Evaluation (APACHE) II scores from 11.12 to 9.19 (*p* < 0.01 for all) [25]. In a single-center, randomized, double-blinded, prospective trial in 60 patients with severe COVID-19, significant improvements in the rates of septic shock and mechanical ventilation, as well as the proportion of patients severely affected, the duration until the main clinical symptoms improved (*p* < 0.05 for all), and the lengths of ICU hospitalization (*p* < 0.01), were observed for the XBJ group (routine medication + XBJ) after 14 days of treatment, compared with the control group (routine medication + saline) [26]. A large-scale survey involving 31,913 hospitalized patients indicated that the incidence of adverse drug reactions (ADRs) to XBJ was at the occasional level (0.3%); most of these reactions were mild or nonserious [27]. Another analysis of data from the Hospital Information System indicated that the ADRs to XBJ were mainly correlated with age, dosage, vehicle type, and drug combination [28]. A recent investigation by our group suggested that the herbal compounds in XBJ have a low potential to participate in therapeutic pharmacokinetic interactions with antibiotics when coadministered with XBJ in sepsis care [29].

Many pharmacological studies have suggested that the antisepsis action of XBJ is correlated with the modulation of the host response, i.e., inhibiting the uncontrolled release of inflammatory mediators, relieving an early overabundant innate immune response and potentially cumulative immunosuppression, attenuating crosstalk between inflammation and coagulation, protecting endothelial cells, and maintaining the physiologic functions of vital organs [30,31,32,33]. Zhou et al. identified four biological functional actions of XBJ in sepsis: the regulation of inflammation, immune activity, cell apoptosis, and coagulation [34]. XBJ can significantly alleviate liver injury in cecal ligation and puncture (CLP) rats via downregulating tumor necrosis factor-α (TNF-α) and interleukin 6 (IL-6) expression while upregulating IL-10 expression and promoting the suppression of cytokine signaling 1 (SOCS1) expression [35]. XBJ played a protective role in methicillin-resistant *Staphylococcus aureus* (MRSA)-challenged mice by downregulating the inflammatory response (IL-6, TNF-α, IL-1β, and IL-12) and signaling pathways (NF-κB, MAPK, and PI3K/Akt) activated by Pam3CSK4 (a synthetic tripalmitoylated lipopeptide mimicking bacterial lipoproteins) [36]. A study based on a sepsis rat model indicated that adding XBJ to antibiotics could improve renal perfusion and oxygenation and suppress renal inflammation, as well as ameliorate kidney dysfunction [37]. A rat- and cell-based study indicated that XBJ may improve pulmonary vascular barrier function by upregulating claudin-5 expression in a rat model with acute lung injury (ALuI) [38]. A GC/MS-based metabonomics approach revealed that XBJ reduced multiorgan dysfunctions in septic rats and increased their survival rate: serum biochemistry indicators including blood urea nitrogen (BUN), creatinine (Cr), alanine aminotransferase (ALT), and aspartate aminotransferase (AST); cytokines (TNF-α and IL-6); and morphologic changes all decreased [39].

XBJ may improve the clinical symptoms and alleviate the disease severity of COVID-19. By using network pharmacology and molecular docking analysis approaches, the active ingredients, potential molecular targets, and mechanisms of XBJ have been investigated [40,41]. Similarly, to explore the multicomponent, multipathway, and multitarget mechanisms of XBJ in sepsis, a drug–target–pathway network and a drug–ingredients–targets–disease network of XBJ were constructed by Zuo et al. and Feng et al., respectively, to identify major active ingredients, targets, and signaling pathways [42,43].

XBJ is a chemically complex herbal injection; more than 100 constituents, including Honghua flavonoids, Chishao monoterpene glycosides, Danshen catechols, Chuanxiong/Danggui phthalides, and other types of constituents, have been detected and characterized in XBJ [29,44]. Additionally, several analytical assays have been developed for the quantification of the multiple constituents in XBJ [44,45,46,47,48]. Based on the comprehensive chemical composition analysis of XBJ, the human pharmacokinetics of XBJ (by dosing with labeled doses) were systematically investigated by Li et al., and the disposition of major circulating XBJ compounds was well characterized with supportive rat studies and in vitro metabolism and transport studies [29,49,50,51]. Accordingly, 13 major circulating XBJ compounds originating from the five component herbs were identified, i.e., hydroxysafflor yellow A from Honghua; paeoniflorin, oxypaeoniflorin, and albiflorin from Chishao; senkyunolide I, senkyunolide I-7-*O*-â-glucuronide, senkyunolide G, and ferulic acid from Chuanxiong and Danggui; tanshinol, 3-*O*-methyltanshinol, protocatechuic acid, salvianolic acid B, and 3-*O*-methylsalvianolic acid B from Danshen. Among these compounds, senkyunolide I-7-*O*-â-glucuronide, 3-*O*-methyltanshinol, protocatechuic acid, and 3-*O*-methylsalvianolic acid B are the in vivo metabolites of senkyunolide I, tanshinol, protocatechuic aldehyde, and salvianolic acid B, respectively; the unchanged compound protocatechuic aldehyde could not be detected in human plasma samples [29,49,50,51]. Several other research groups also measured circulating herbal compounds in their unchanged forms in rats receiving XueBiJing based on developed bioanalytical assays [52,53,54,55]. Zuo et al. investigated the tissue distributions of several bioactive compounds in rats after they intravenously received XBJ, and the levels of exposure to four compounds (i.e., hydroxysafflor yellow A, paeoniflorin, ferulic acid, and benzoylpaeoniflorin) were found to be high in the kidneys, liver, stomach, and intestines [52]. Hydroxysafflor yellow A, despite its poor membrane permeability, could partly cross the damaged blood–brain barrier in patients with traumatic brain injury after the intravenous administration of XBJ [56].

**Figure 1:**
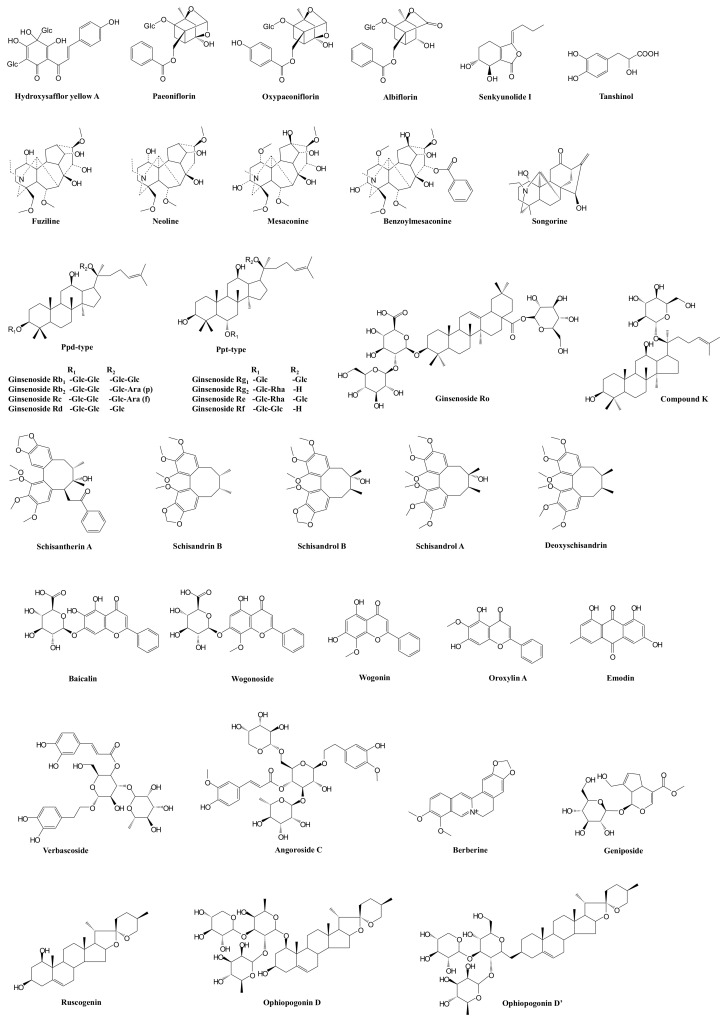
Chemical structures of bioactive and bioavailable herbal compounds from antisepsis Chinese herbal medicines.

The antisepsis-related activities—i.e., anti-inflammatory, anticoagulant, endothelium-protective, immune-regulatory, antioxidant, and organ-protective activities—of the aforementioned unchanged circulating compounds from XBJ, based on animal or cellular studies, have been widely reported [57,58,59,60,61,62,63,64]. However, the experimental doses or concentrations of the test XBJ compounds were poorly related to their systemic exposure levels. Therefore, the antisepsis-related activities of the major pharmacokinetically identified circulating compounds were systematically evaluated at the concentrations of their systemic exposure levels after dosing XBJ in in vitro studies and for individual doses of XBJ in a CLP rat study. Finally, six XBJ compounds (hydroxysafflor yellow A, paeoniflorin, oxypaeoniflorin, albiflorin, tanshinol, and senkyunolide I; Figure 1) were identified to be the material basis of XBJ: the survival rate of CLP rats receiving the intravenous injection of the combination of the six XBJ compounds proved to be comparable to that of CLP rats receiving XBJ. The survival rates of both groups were significantly lower than that of CLP control rats receiving 0.9% saline (*p* < 0.05; pending publication). Table 1 lists some potential target pathways of the bioavailable and bioactive XBJ compounds.

#### 2.1.2. ShenFu Injection

The ShenFu injection (SF), derived from the ShenFu decoction, is a standardized intravenous herbal medicine prepared from a combination of *Panax ginseng* steamed root (Hongshen) and processed *Aconitum carmichaelii* lateral root (Fuzi). It is manufactured by Ya’an Sanjiu Pharmaceutical (Ya’an, Sichuan Province, China) with an NMPA drug ratification number of GuoYaoZhunZi-Z51020664. As an emergency medicine, SF is commonly applied in combination with chemotherapy to fight against shock, acute myocardial dysfunction, chronic congestive heart failure, etc. [65,66,67,68]. By supplying qi and strengthening yang in terms of traditional Chinese medicine (TCM) theory, SF is widely used for the treatment of Yin-yang Jutsu syndrome and severe deficiency syndromes with signs of hidrosis, mental exhaustion, breathlessness, uroclepsia, a weak pulse, etc. [15].

For septic shock patients, the TCM syndrome score facilitates the evaluation of the effect of the TCM syndrome and the construction of a treatment plan. Based on this strategy, the combination of SF with standard bundle therapy significantly improved patients’ circulation, tissue perfusion, and coagulation function, as well as inflammation reactions [69]. Adding SF to conventional therapy could increase patients’ mean arterial pressure (MAP), normalize the heart rate, clear serum lactate, and reduce the mortality of patients, thus reducing the occurrence of septic shock and the need for resuscitation [70,71]. Based on a systematic review and meta-analysis of randomized controlled trials, compared with standard therapy, the addition of SF showed a trend of decreasing 28-day mortality (*p* = 0.17) only for the septic shock patients, with 4.5 mmol/L ≤ mean arterial lactate level < 7 mmol/L and with a yang-qi deficiency (a TCM syndrome) [72]. A multicenter, randomized, controlled, open-labeled trial carried out in 210 patients with septic shock in China suggested that adding SF to the conventional treatment further improved the 7-day survival rate (83.3% versus 54.5%, *p* = 0.034) in patients with impaired lactate clearance (≥4.5 mmol/L) [73]. SF can enhance the cellular immunity of patients with septic shock by increasing CD4+ and CD8+ T cells in the peripheral blood and upregulating human leukocyte antigen-DR (HLA-DR) expression in monocytes. SF was also found to restore ex vivo monocytic TNF-α and IL-6 proinflammatory cytokine release in response to endotoxins. In addition, patients in the SF group (*n* = 78) showed better clinical outcomes than those in the placebo group (*n* = 79) in terms of the APCHE II score (13.2 ± 7.6 vs. 16.9 ± 8.8; *p* = 0.034), the Marshall score (6.8 ± 2.6 vs. 8.5 ± 3.3; *p* = 0.01), the duration of vasopressor use (2.5 ± 1.5 vs. 3.7 ± 1.7 days; *p* = 0.008), and the length of ICU stay (10.5 ± 3.2 vs. 12.2 ± 2.8 days; *p* = 0.012) [74]. A clinical investigation in 89 elderly patients with severe pneumonia indicated that compared with the control group, the serum levels of TNF-α, IL-6, and IL-8 after 7-day treatment with SF significantly decreased (*p* < 0.05), while the serum level of IL-10 obviously increased (*p* < 0.05). The APACHE II score was significantly lower than that before the treatment (it decreased from 17.4 ± 3.2 to 8.6 ± 3.5; *p* < 0.05), whereas the application time for vasoactive drugs, the time of mechanical ventilation, and the duration in the ICU were notably shortened (*p* < 0.05) [75]. 

SF has been widely used in clinical patients since it became available on the market in 1987, and all the reported side effects are mild [152]. In safety monitoring for SF involving 30,106 patients, adverse drug events (ADEs) occurred in only 114 patients, and ADRs occurred in only 23 patients, showing a rare-level incidence rate of 0.076% [153].

Gastrointestinal mucosal injury and gastrointestinal dysfunction in patients with sepsis indicate the aggravation of sepsis or that the prognosis is worsening [154,155]. Xing et al. reported the protective effect of SF on the intestinal mucosal barrier in a rat model of sepsis, with intestinal mucosal disruption accompanied by accelerated apoptosis of the epithelial cells, leading to bacterial translocation and progression to multiple organ dysfunction [156,157]. The rats administered a low (5 mL/kg) or high (20 mL/kg) dose of SF showed lower mortality, lower intestinal mucosal injury, and lower serum TNF-α and IL-6 levels (*p* < 0.05), as well as higher secretory immunoglobulin A (sIgA) levels and CD3 and γδT cell numbers (*p* < 0.01), than the model group, in a dose-dependent manner [158]. SF also exerted a protective effect on lipopolysaccharide (LPS)-induced septic shock in rabbits by increasing MAP; decreasing serum lactate dehydrogenase (LDH) and AST levels; improving the heart, liver, and kidney morphology of LPS-induced rabbit models with septic shock [159]. SF attenuated the inflammation and apoptosis induced by LPS in rats via downregulating the mitogen-activated protein kinase (MAPK) and extracellular regulated protein kinase signaling pathways [160]. SF suppressed sepsis-induced myocardial apoptosis and injury by upregulating the protein expression of B-cell lymphoma 2; downregulating that of BH3 interacting-domain death agonist, truncated-Bid, and caspase-9 (*p* < 0.05); and alleviating mitochondrial damage [161]. The levels of several biomarkers (TNF-α, the ileal malondialdehyde level, the apoptosis index for ileal mucosal epithelial cells, and the Bax protein level) were significantly higher in the CLP group than in the sham group (*p* < 0.01 or *p* < 0.05), while some others (the serum level of IL-10, Bcl-2/Bax ratio, Bcl-2 protein level, and occludin protein level) were significantly lower. Both low-dose (5 mL/kg) and high-dose (10 mL/kg) SF significantly ameliorated these changes (*p* < 0.01 or *p* < 0.05) in a dose-dependent manner [162]. SF also dose-dependently prevented MAP drop, relieved lung damage, and increased the survival rate in the rat model of endotoxin shock, perhaps through inhibiting the high-mobility group protein B1 (HMGB1)–nuclear factor κB (NF-κB) pathway, thus preventing a cytokine storm [163].

SF contains multiple herbal constituents, including ginsenosides (originating from Hongshen), aconitum alkaloids (from Fuzi), and organic acids (mainly from Fuzi) [164,165]. Yang et al. detected 44 herbal constituents (i.e., 19 ginseng saponins, 1 panaxytriol, 1 5-hydroxymethylfurfural, and 23 trace diterpene alkaloids) in SF and quantified 24 major ginsenosides and alkaloids. The total concentrations of saponins and alkaloids were 676–742 μg/mL and 3–7 μg/mL, respectively, in five batches of SF samples. The ginsenosides Rb_1_ and Rg_1_ were higher in content than other constituents, i.e., 176.4 ± 1.4 μg/mL (159.0 μmol/L) and 120.0 ± 1.3 μg/mL (149.9 μmol/L), respectively. In addition, a high batch-to-batch quality consistency for SF samples was observed [166]. In addition to hydrophobic aconite alkaloids and ginsenosides, another 157 hydrophilic compounds (154 compounds identified as amino acids, nucleosides, organic acids, carbohydrates, etc.; 3 compounds unknown) were detected in SF by Song et al. In addition, 40 primary hydrophilic and hydrophobic ingredients (11 ginsenosides, 9 aconite alkaloids, 11 amino acids, and 9 nucleosides) were quantitatively or semi-quantitatively analyzed, and the mean contents of the ginsenosides Rb_1_ (129.3 μg/mL; 116.6 μmol/L) and Rg_1_ (97.1 μg/mL; 121.3 μmol/L) in SF were also found to be much higher than those of the aconite alkaloids songorine (0.13 μg/mL; 0.36 μmol/L), benzoylmesaconine (2.43 μg/mL; 4.12 μmol/L), benzoylhypaconine (0.60 μg/mL; 1.05 μmol/L), and hypaconitine (0.02 μg/mL; 0.03 μmol/L) [167]. On the basis of in vitro and in silico studies, Li et al. identified some NF-κB inhibitors for counteracting inflammation in SF such as 20(*S*)-protopanaxadiol type (ppd-type) glycosides (ginsenosides Rb_1_, Rb_2_, Rb_3_, Rc, and Rd), 20(*S*)-protopanaxatriol type (ppt-type) glycosides (ginsenosides Rg_1_, Rg_2_, Re, Rf, and F_1_), diester-type alkaloids (fuziline and neoline), and aconine derivatives (mesaconine and benzoylmesaconine) [168].

After intravenously administrating SF to rats at a dosage of 5.0 mL/kg, the systemic exposure to the ppd-type ginsenosides Rb_1_, Rc, and Rb_2_ in rat plasma (64.3 ± 28.1, 60.5 ± 26.9, and 41.2 ± 18.8 μmol·h/L, respectively) was much higher than that to the ppt-type ginsenosides Rf and Rd (5.47 ± 4.12 and 2.97 ± 2.13 μmol·h/L, respectively), which is probably because Rb_1_, Rc, and Rb_2_ (0.59, 0.53, and 0.39 μmol/kg, respectively) had higher contents than Rf and Rd (0.12 and 0.86 μmol/kg, respectively) and also had much longer half-life *t*_1/2_ (19.3 ± 6.4, 29.5 ± 22.9, and 35.6 ± 30.7 h, respectively) than Rf and Rd (4.21 ± 3.68 and 8.49 ± 5.20 h, respectively) [169]. The short *t*_1/2_ of ppt-type ginsenosides was mainly attributed to transporter-mediated rapid biliary excretion [91,170]. After a single intravenous bolus of SF at 2 mL in Wistar rats (10 mL/kg), the *t*_1/2α_ of Rd, Rg_1_, Rb_1_, Ro, Rc, and Rb_2_ was 0.32 ± 0.25, 0.11 ± 0.04, 0.30 ± 0.24, 0.11 ± 0.02, 0.24 ± 0.19, and 0.23 ± 0.12 h, respectively; the *t*_1/2β_ was 10.25 ± 0.39, 21.31 ± 3.64, 13.95 ± 2.56, and 15.06 ± 1.54 h, respectively; the systemic exposure to Rd, Rg_1_, Rb_1_, Ro, Rc, and Rb_2_ in rats was 879.8 ± 137.3, 8.07 ± 1.56, 1742.4 ± 343.6, 6.77 ± 0.58, 1001.5 ± 125.2, and 1533.7 ± 229.0 μmol·h/L, respectively [171]. Ginsenosides (Rg_1_, Rb_1_, and Rc) and aconitum alkaloids (benzoylmesaconine and fuziline) were detected in dog plasma after the intravenous drip administration of 2, 4, or 8 mL/kg of SF. The maximum plasma concentrations (*C*_max_) of the five analytes were achieved at the point of infusion completion after the single-dose administration of SF, i.e., *T*_max_ as 1 h. The plasma *t*_1/2_ was short for benzoylmesaconine and fuziline (approximately 5 and 2 h, respectively); this relative rapid elimination makes them relatively safe for clinical use due to the two alkaloids’ low toxicity. Similarly, the elimination of the ppt-type ginsenoside Rg_1_ was also quick (*t*_1/2_, less than 0.5 h), while the elimination of the ppd-type ginsenosides Rb_1_ and Rc was much slower (70 and 90 h, respectively), which facilitates maintaining effective systemic exposure levels and achieving better therapeutic effects. After the intravenous infusion of SF in beagles, the plasma concentrations of the five analytes all increased proportionally over the dosage range of 2–8 mL/kg [172]. The chemical structures of major circulating SF compounds are shown in Figure 1, and their potential action target pathways are summarized in Table 1.

### 2.2. Chinese Herbal Prescriptions

#### 2.2.1. ShengMai Formula

The ShengMai formula (SMF), which was first recorded in Yi Xue Yuan Li, consists of *P. ginseng* root (Renshen), *Ophiopogon japonicus* root (Maidong), and *Schisandra chinensis* fruit (Wuweizi) with a dosage proportion of 5:3:1.5. It is normally prepared as ShengMai powder (SMP; or ShengMai san, SMS), ShengMai yin (SMY), ShengMai injection (SMI), etc., for clinical use. SMF is a classic tonic prescription for the treatment of tuberculosis, chronic bronchitis, cough due to neurasthenia, and heart failure [173]. SMI, an intravenous dosage form of SMF, is used to treat acute myocardial infarction, cardiogenic shock, toxic shock, hemorrhagic shock, coronary heart disease, endocrine disorders, and other diseases due to a deficiency of qi and yin, with low toxicity [174,175]. SMI is highly recommended for use in combination with antibiotics for community-acquired pneumonia in clinical guidelines [16]. A meta-analysis including 17 randomized controlled trials (RCTs) and 860 patients with septic shock suggested that adding SMI to conventional Western medicine treatment further reduced the number of ineffective shock treatments (*p* < 0.0001) and reduced the blood lactate concentration at 12 h (*p* < 0.001), 24 h (*p* < 0.0001), and 72 h (*p* = 0.002) [176].

SMI protects multiple organs by regulating immunity, inflammation, apoptosis, and energy metabolism. SMI also protected the intestinal mucosal barrier of mice mainly through regulating the NF-κB–pro-inflammatory factor–myosin light-chain kinase (MLCK)–TJ cascade. Decreasing trends for inflammatory factors including interferon-γ (IFN-γ), TNF-α, and IL-2 were observed in the sera of mice receiving SMI at 1.5 mL/kg. The content of occludin increased and MLCK protein decreased in SMI-treated mice compared with the endotoxemia mouse model group (*p* < 0.05 or *p* < 0.01) [177]. SMI could induce myocardial mitochondrial autophagy via the caspase-3/Beclin-1 axis to protect myocardial mitochondria in septic mice [178]. A study by Chai et al. on CLP rats suggested that the regulation of taurine and taurine metabolism, as well as arginine and proline metabolism, etc., could be the key mechanism in the treatment of sepsis [179].

Zheng et al. recently reviewed, in Chinese, the material composition, preclinical pharmacokinetic, and pharmacodynamic studies of SMI [180]. Several research groups have analyzed the chemical compositions of SMF preparations and identified the main constituents as ginsenosides (originating from *P. ginseng*), steroidal saponins (from *O. japonicus*), lignans (from *S. chinensis*), and flavonoids (mainly from *O. japonicus*). Using LC-IT-TOF/MS and a diagnostic fragment-ion-based extension strategy, Zheng et al. detected and identified more than 30 ginsenosides and 20 lignans from SMI [181]. Zhao et al. identified or partially characterized 87 herbal compounds in SMI and selected 6 bioactive constituents (four ginsenosides (i.e., Rg_1_, Re, Rb_1_, and Rd) and 2 lignans (i.e., schisandrol A and schisandrol B) with high content levels as quality markers (Q-markers). The total content range for these selected Q-markers in 10 batches of SMI was 13.8–22.5 mg/mL [182]. Wu et al. identified 92 compounds (i.e., 49 ginsenosides, 31 lignans, 5 steroidal saponins, and 7 homoisoflavanones) in SMP and discovered a class of 25-hydroxyginsenosides for the first time [183]. In a study by Cheng et al., 10 compounds (the ginsenosides Rb_1_, Rb_2_, Rc, Rd, Re, Rg_1_, and Rh_1_; compound K; ophiopogonin D; and schisandrol A) were measured in SMP, and the contents of these herbal constituents were found to vary by up to several hundredfolds among five pharmaceutical manufacturers [184]. In their study, Zheng et al. selected eight compounds (the ginsenosides Rf, Rb_1_, Rg_2_, and Rb_2_; schisandrol A; schisandrol B; methylophiopogonanone A; and schisandrin B) as Q-markers to evaluate the batch-to-batch consistency of SMF; ginsenoside Rb_1_, ranging from 2046.1 μg/g (1.84 μmol/g) to 5975.8 μg/g (5.39 μmol/g), was found to be the dominant component in SMF, followed by ginsenoside Rg_2_ (838.3–2091.64 μg/g; 1.07–2.66 μmol/g) and ginsenoside Rb_2_ (567.2–1989.9 μg/g; 0.53–1.84 μmol/g). The batch-to-batch chemical variation among 10 batches of SMF ranged from 27.9% (for ginsenoside Rf) to 113.95% (for schisandrol B) [185]. Li et al. established a seven-marker-based quality standard to quantify seven ginsenosides (i.e., the ginsenosides Rf, Rd, Rc, Re, Rb_1_, Rb_2_, and Rg_1_) in SMI, which was then used to evaluate the quality consistency of 22 batches of SMI [186]. Li et al. detected 62 compounds in SMI and established a quantitative assay for the determination of 21 main components, including 14 saponins, 6 lignans, and 1 pyranoglucoside, and found the contents of these 21 components to vary widely amongst 10 batches [187].

Lu et al. established a TCM–components–core targets–key pathway network platform to investigate the mechanism of SMI’s effects in sepsis. SMI was found to mainly affect several signaling pathways, suggesting that SMI could regulate immunity, inflammation, apoptosis, and energy metabolism for the protection of multiple organs. Gene ontology (GO) enrichment analysis further indicated that the bioactive SMI constituents altered the pathophysiology of sepsis through the regulation of various biological processes [188]. SMP protected against I/R-induced blood–brain barrier (BBB) dysfunction by significantly upregulating ZO-1 and claudin-5 under oxygen-glucose deprivation/reoxygenation (OGD/R), as well as reducing matrix metalloproteinase 2/9 (MMP-2/9) levels and the phosphorylation of myosin light-chain (MLC) through the ROCK/cofilin signaling pathway [189].

Zhan et al. developed and validated a sensitive LC-MS/MS method for the simultaneous quantification of 11 SMI compounds in rat serum and applied it to a pharmacokinetic study in rats after a single intravenous administration of SMI. The 11 constituents were ppt-type ginsenosides (i.e., the ginsenosides Rg_1_, Re, Rf, and Rg_2_), ppd-type ginsenosides (i.e., the ginsenosides Rb_1_, Rd, and Rc), ophiopogonin (ophiopogonin D), and lignans (i.e., schisandrol A, schisandrol B, and schisandrin B) [190,191]. A total of 30 compounds (23 prototype components and 7 metabolites) were detected and characterized in the plasma of rats after they received SMS (8 g/kg) [192,193]. Further, ppt-type ginsenosides were eliminated rapidly through urinary, biliary, and fecal excretions (plasma *t*_1/2â_, 0.60–0.82 h; MRT, 0.22–0.46 h), whereas the ppd-type ginsenosides Rb_1_, Rd, and Rc exhibited slow elimination through biliary and urinary excretions (MRT, 23.0‒28.6 h). Ophiopogonin D was mainly excreted in bile in the metabolized forms. Schisandrol A, schisandrol B, and schisandrin B, with low contents in SMI, were found to be eliminated quickly (plasma *t*_1/2â_, 0.51‒1.98 h; MRT, 0.51‒2.50 h) and accumulated in these tissues. Lignans were mainly excreted in their metabolized form, as indicated by the very low biliary, urinary, and fecal excretion of the unchanged forms [190,191]. SMI, within the concentration range of 30% (volume percentage), showed an inhibitory effect on the activities of CYP1A2, CYP2B6, CYP2C8, CYP2C9, CYP2C19, CYP2D6, and CYP3A4, with IC_50_ values of 6.12%, 2.72%, 10.00‒30.00%, 14.31%, 12.96%, 12.26%, and 3.72%, respectively, and had an inhibitory effect on the activities of the transporters MDR1, BCRP, and organic anion transporting polypeptide (OATP)1B1, with IC_50_ values of 0.15%, 0.75%, and 2.03%, respectively. This suggested a high risk of drug interactions of SMI when clinically combined with the use of the transporters MDR1 and BCRP substrate [194]. SMS selectively suppressed intestinal, but not hepatic, nifedipine oxidation (a CYP3A marker reaction) activity in a dose- and time-dependent manner. Three-week SMS treatment decreased the maximal velocity of intestinal nifedipine oxidation by 50%, while the CYP3A protein level remained unchanged; among the SMS component herbs, the decoction of *Ophiopogonis Radix* decreased the intestinal nifedipine oxidation activity [195]. Based on an inhibition kinetic investigation of various UGT isoforms, ophiopogonin D was found to noncompetitively inhibit UGT1A6 (*K*_i_, 20.6 μmol/L) and competitively inhibit UGT1A8 (40.1 μmol/L); ophiopogonin D’ noncompetitively inhibited UGT1A6 (5.3 μmol/L) and UGT1A10 (9.0 μmol/L); and ruscorectal competitively inhibited UGT1A4 (0.02 μmol/L) [106]. The ginsenoside Rg_1_, ophiopogon D’, and schisandrin A are potential inhibitors of sodium taurocholate co-transporting polypeptide (NTCP) and probably interact with NTCP-modulating clinical drugs. The ginsenoside Re and schisandrin B are potential NTCP substrates, and their NTCP-mediated uptake could be inhibited by other ingredients in SMF [100]. The ginsenosides Rb_2_, Rc, Rg_2_, Rg_3_, Rd, and Rb_1_ are P-gp substrates, and *Schisandra Lignans* extract (SLE) was found to significantly enhance the uptake and inhibit the efflux ratio of the ginsenosides Rb_2_, Rc, Rg_2_, Rg_3_, Rd, and Rb_1_ in Caco-2 and L-MDR1 cells. Additionally, a rat study showed that a single dose and multiple doses of SLE at 500 mg/kg could significantly increase the AUC_0–∞_ of Rb_2_, Rc, and Rd without affecting the *t*_1/2_ [196]. The chemical structures of the major circulating SM compounds are shown in Figure 1, and their potential action target pathways are summarized in Table 1.

#### 2.2.2. Qingwen Baidu Decoction

The Qingwen Baidu decoction (QWBD), first recorded in the book Yi Zhen Yi De of the Qing Dynasty, is a famous anti-epidemic TCM prescription [197]. It has been used for the treatment of summer-heat syndrome in epidemic febrile disease in China for many years [198]. It consists of *Rehmannia glutinosa* root (Dihuang), *Rhinoceros unicornis* horn (Xijiao), *Coptidis chinensis* rhizome (Huanglian), *Gardenia jasminoides* fruit (Zhizi), *Platycodon grandiflorum* root (Jiegeng), *Scutellaria baicalensis* root (Huangqin), *Anemarrhena asphodeloides* rhizome (Zhimu), *Paeonia lactiflora* root (Chishao), *Scrophularia ningpoensis* root (Xuanshen), *Forsythia suspense* fruit (Lianqiao), *Lophatherum gracile* stem and leaf (Danzhuye), *Glycyrrhiza uralensis* root and rhizome (Gancao), *Paeonia suffruticosa* root cortex (Danpi), and *Gypsum Fibrosum* (Shigao) [197]. Wang et al. proposed a protocol for a systematic review and meta-analysis of QWBD for sepsis [198]. Recently, Wen et al. reviewed the potential therapeutic effect of QWBD against COVID-19 [197]. A promising effect was observed upon adding QWBD to conventional Western medical treatment for 18 patients with pulmonary infection, by Sun et al. [199].

Yu et al. found that QWBD produced anti-inflammatory effects by altering the levels of inflammatory mediators in sepsis rats [200]. Based on a network pharmacology study, QWBD was found to exert antisepsis actions by regulating protein phosphorylation, the cell response to cytokine stimulation, cell proliferation, the inflammatory response, the transmembrane receptor protein tyrosine kinase signaling pathway, and cytokine-mediated signaling pathways [201]. Although the phytochemistry of the component herbs Dihuang [202], Xijiao [203], Huanglian [204], Zhizi [121,205], Jiegeng [206], Huangqin [207], Zhimu [208], Chishao [209], Xuanshen [210], Lianqiao [211], Danzhuye [212], Gancao [213], Danpi [214], and Shigao (CaSO_4_·2H_2_O) have been widely reported, chemical composition studies of QWBD are limited. A total of 21 compounds from 11 component herbs were detected in QWBD and characterized, among which 15 analytical markers were selected for the quality evaluation of QWBD: baicalin (content level, 563.1–852.8 μg/g or 1.26–1.91 μmol/g), wogonoside (64.9–106.8 μg/g or 0.14–0.23 μmol/g), geniposidic acid (10.1–21.1 μg/g or 0.03–0.06 μmol/g), oxypaeoniflorin (18.2–25.3 μg/g or 0.04–0.05 μmol/g), genipin 1-β-D-gentiobioside (25.7–60.7 μg/g or 0.047–0.11 μmol/g), geniposide (131.9–396.7 μg/g or 0.34–1.02 μmol/g), paeoniflorin (201.2–305.5 μg/g or 0.42–0.64 μmol/g), mangiferin (50.6–79.2 μg/g or 0.12–0.19 μmol/g), swertiajaponin (17.9–58.7 μg/g or 0.037–0.12 μmol/g), acteoside (106.8–143.8 μg/g or 0.17–0.23 μmol/g), forsythoside A (124.2–261.6 μg/g or 0.20–0.42 μmol/g), berberine hydrochloride (156.2–654.1 μg/g or 0.42–1.76 μmol/g), paeonol (6.24–18.5 μg/g or 0.038–0.11 μmol/g), harpagoside (3.21–14.1 μg/g or 0.006–0.029 μmol/g), and glycyrrhizic acid (46.9–14.1 μg/g or 0.057–0.16 μmol/g). QWBD exhibited potent anti-inflammatory effects in a dose-dependent manner based on a study in zebrafish inflammatory models. The mechanism may be related to the activation of the NF-κB and signal transducer and activator of transcription (STAT)3 signaling pathways [215]. The high (38 g/kg) and medium (19 g/kg) doses of QWBD showed significantly potent anti-inflammatory effects and reduced the pulmonary edema caused by ALuI in rats (*p* < 0.05). HPLC-DAD-ESI-MS^n^ combined with PCA indicated that verbascoside and angoroside C could reduce pulmonary edema. In addition, five compounds (i.e., galloylpaeoniflorin, pentagalloylglucose, mudanpioside C, ethyl gallate, and harpagoside) reduced the total cells of activated polymorphonuclear leukocytes and their infiltration for the treatment of ALuI [216]. Pharmacokinetic studies of QWBD are scarce. The chemical structures of some representative QWBD compounds are shown in Figure 1, and their potential action target pathways are summarized in Table 1.

#### 2.2.3. Xuanbai Chengqi Decoction

The Xuanbai Chengqi decoction (XBCQ), first recorded in Wen Bing Tiao Bian, consists of *Rheum palmatum* rhizome and root (Dahuang), *Gypsum Fibrosum* (Shigao), *Prunus armeniaca* seed (Kuxingren), and *Trichosanthes kirilowii* fruit (Gualou). XBCQ “improved static/dynamic lung compliance” but also “reduced the complication incidence in patients with ARDS” [217]. XBCQ is also the basic formulation for Qifen syndrome in COVID-19 [218]; in the critical stage, XBCQ is considered to reduce phlegm and clear heat [219]. A meta-analysis involving 11 RCTs and 992 participants indicated that XBCQ combined with Western medicine provided a better benefit than Western medicine alone to patients with severe pneumonia with the symptom pattern of phlegm–heat obstructing lungs in terms of the total effective rate (RR = 1.23; 95% CI (1.16, 1.30)), clinical pulmonary infection score (CPIS; MD = −2.02; 95% CI (−2.42, −1.63)), APACHE II (MD = −6.81; 95% CI (−8.26, 5.37)), mechanical ventilation time (MD = −101.41; 95% CI (−140.47, −62.34)), and lactic acid content in arterial blood (MD = −2.41; 95% CI (−2.64, −2.18)) [220]. For patients meeting the ARDS diagnostic criteria, the static lung compliance and dynamic lung compliance in the treatment group (adding XBCQ to conventional treatment), at 48 and 72 h after treatment, were significantly higher than in the control group (conventional treatment), and the plateau pressure, peak airway pressure, and positive end-expiratory pressure (PEEP) were significantly lower than before treatment [217].

In recent years, a growing body of evidence is showing that gut microbiota dysbiosis and overwhelming inflammation play an essential role in cell dysfunction and organ failure [221,222]. Gut microbiota dysbiosis can alter the dominant bacterial genera *Clostridia* and *Enterococcaceae* [223], and result in the loss of vital bacterial genera that produce short-chain fatty acids in healthy human beings, such as *Prevotella* and *Ruminococcaceae* [224]. At the initial hyper-inflammatory stage of sepsis, accompanied by alterations in the structural and functional stability of gut integrity, bacteria and their products translocate via the mesenteric lymph node or portal venous blood and finally cause SIRS, ARDS, and MODS [222,225]. Dickson et al. discovered that the lung microbiome in patients with ARDS and sepsis was enriched with enteral bacteria. They also revealed a shared mechanism of pathogenesis on the basis of the close association between the relative abundance of enteral *Bacteroides* spp. and the serum level of TNF-á in patients with lethal diseases [226]. In a study by Mu et al., XBCQ exhibited protective effects in CLP rats by modulating the gut microbiota, restoring the gut barrier, and downregulating inflammatory responses [227]. Based on a network pharmacology study, the regulation by XBCQ of the PI3K/mTOR/HIF-1α/VEGF signaling pathway was proposed to be a crucial mechanism of the protective effect of XBCQ in the treatment of ALuI [228].

**Figure 2:**
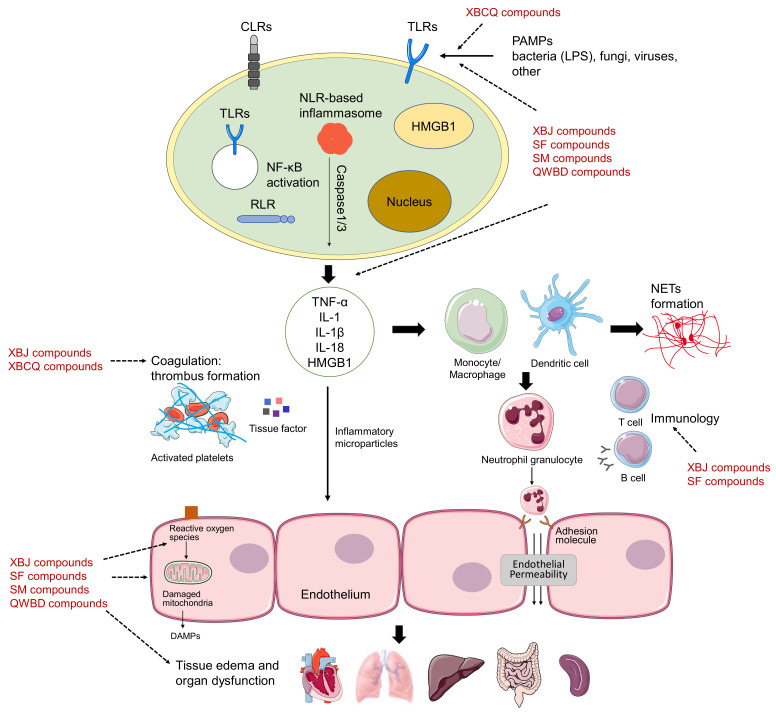
Potential mechanism of antisepsis actions of the five CHM based on pathophysiologic processes involved in sepsis. The dash line arrows indicate proposed action targets or signaling pathways that the five CHM probably involve; the solid line arrows indicate cascade mechanism of pathophysiology in sepsis. CLRs, C-type lectin receptors; TLRs, toll-like receptors; NLR, nucleotide binding domain and leucine-rich-repeat-containing proteins; RLR, Retinoic acid-inducible gene-like receptors; HMGB1, high mobility group B-1; PAMPs, pathogen-associated molecular patterns; LPS, lipopolysaccharide; TNF-α, tumor necrosis factor-α; IL-1, interleukin 1; IL-1β, interleukin 1β; IL-18, interleukin 18; DAMPs, damage-associated molecular-pattern molecules.

Phytochemistry studies of XBCQ are limited, although the chemical compositions of the component herbs Dahuang [229], Kuxingren [230], and Gualou [231] and the mineral medicine Shigao (CaSO_4_·2H_2_O) have been widely investigated and defined. Pharmacokinetic studies of XBCQ are also limited. Emodin (originating from Dahuang; Figure 1) is the major constituent of XBCQ, and its potential target pathways are listed in Table 1. The potential mechanism of antisepsis actions of the five CHM based on pathophysiologic processes involved in sepsis is shown in Figure 2.

### 2.3. Other Herbal Medicines

Xing et al. summarized TCM combination therapies to treat septic acute gastrointestinal injury patients [232]. Compared with the control group, the 28-day mortality and gastrointestinal injury in the TCM-intervention group were significantly reduced (*p* < 0.05), as were the durations of mechanical ventilation and ICU stays (*p* < 0.05). Wang et al. found that the Sini decoction could restore microbial richness and abundance, reestablish the balance of intestinal flora, and thus ameliorate sepsis-related symptoms and pathology in CLP mice [233]. The Sini decoction was also found to ameliorate adrenal stress responses by downregulating TLR4 expression in adrenal tissue, demonstrating its promise for the prevention of adrenal insufficiency in prolonged sepsis [234]. The Fangji Fuling decoction inhibited the inflammatory and apoptotic response and further alleviated LPS-induced acute kidney injury [235]. The Xuefu Zhuyu decoction protected the myocardium in sepsis rats by the inhibition of myocardial cell apoptosis and antioxidation [236], whereas Shengjiang powder produced the same myocardium-protective effect by the downregulation of p38 MAPK phosphorylation [237]. The Xijiao Dihuang decoction was able to improve survival in sepsis via the regulation of the NF-κB and hypoxia-inducible factor-1α signaling pathways [238].

## 3. Discussion and Conclusions

TCM plays an important and distinctive role in the treatment of sepsis in China, especially in the fight against COVID-19. More and more basic scientific research with regard to the pharmacological action, phytochemistry, and pharmacokinetics of TCM is being conducted. Nevertheless, the modernization of TCM still requires considerable work. The clinical efficacy of antisepsis CHM has been partly proven by well-designed and effectively executed clinical trials, meta-analyses aggregating the results of several similarly designed trials, and/or recommendations by authoritative treatment guidelines and expert consensuses. More multicenter, randomized, double-blind, placebo-controlled trials are needed to provide more evidence of clinical efficacy. The physiological and biochemical effects relevant to the antisepsis action of herbal medicines (as well as their component herbs or herbal compounds) have also been widely investigated via animal and/or cellular studies. However, studies extrapolating in vitro to in vivo and the translation of the antisepsis-related pharmacological properties from the laboratory to the clinic are insufficient. Thus, the material bases of most antisepsis CHM and the mechanisms of pharmacological actions have not yet been fully elucidated.

Phytochemistry studies of CHM provide the foundation of pharmacokinetic and pharmacology studies, based on which bioavailable and bioactive herbal compounds are identified, providing the foundation for developing potential drugs derived from CHM. Due to their complexity, the chemical composition analysis of herbal medicines normally requires advanced analytical technology and rich knowledge of phytochemistry. The chemical compositions of some antisepsis herbal injections have already been well defined, but for herbal prescriptions with more complex constituents, especially formulas containing several or more than ten component herbs, their definition is more difficult, and much more research needs to be conducted.

In recent years, the underlying mechanisms of action of antisepsis CHM have been tentatively explored using network pharmacology and molecular docking analysis. Several network pharmacology methods for TCM studies have been developed, mainly to predict the pharmacological actions of herbal compounds and their targets, as well as to screen synergistic multiple compounds from herbal formulas [239]. Bioactive compounds could be discovered and the mechanisms of action of herbal formulas could be tentatively elucidated using network-based methods. However, network pharmacology has its own limitations: (1) it mainly focuses on unchanged compounds (prototype constituents) of herbal medicines rather than their real systemic exposure forms (unchanged and/or metabolized) after the administration of the medicines, and (2) it normally does not incorporate the fluctuation in compounds’ concentrations (systemic exposure levels) over time or the compounds’ reachability in vivo after dosing the medicines. In terms of these two points, pharmacokinetics could act as a powerful complement to network pharmacology studies and provide important information for traditional pharmacology studies.

Pharmacokinetics plays an important role in clarifying the material basis of CHM. Based on comprehensive composition analysis of antisepsis CHM, pharmacokinetics is used to identify herbal compounds with high systemic exposure (bioavailable com pounds) for further antisepsis-related pharmacological studies (i.e., pharmacokinetics provides bioavailable compounds and concentrations). However, pharmacokinetic studies of these antisepsis CHM have been in their infancy for a long time, which is mainly attributed to the limitations of analytical techniques and pharmacokinetic knowledge. In just under a decade, the pharmacokinetic studies of CHM have rapidly developed [92,93,240,241] and the pharmacokinetic studies of XBJ provide a successful example of this [29,49,50,51]. Pharmacokinetic studies have suggested that the material-basis studies of herbal medicines should not only be concerned with the unchanged compounds (prototypes) but also the metabolites. Accordingly, antisepsis-related pharmacological studies, focusing mainly on the significantly bioavailable herbal compounds (in unchanged and/or metabolized forms), facilitate revealing the material bases of antisepsis herbal medicines.

The destruction of the intestinal microbiota is a risk factor for sepsis. In sepsis, the compositions of the intestinal microbiomes of patients are severely affected, which might lead to the development of organ failure. Therefore, the modulation of the gut microbiota and the improvement of intestinal barrier function are expected to be important for the prognosis of sepsis patients [227]. CHMs have been demonstrated to restore microbiota homeostasis, improve intestinal and lung epithelium proliferation, improve intestinal barrier integrity, and suppress hyperimmune reactions [227]. Some types of herbal medicines can regulate the composition and metabolism of the intestinal flora, thereby improving the body’s dysfunction and pathological conditions; for instance, glycosides, flavonoids, alkaloids, phenylpropanoids, and organic acids are known to affect the intestinal flora. The intestinal florae participate in the metabolic transformation of herbs but also transform herbal compounds to improve bioavailability. Flavonoids have certain regulatory effects on the intestinal flora and can be catabolized by microorganisms, causing changes in their bioavailability and activity. Therefore, understanding the roles in regulating intestinal florae is important for clarifying the mechanisms of action of antisepsis herbal medicines.

Based on the current studies of the antisepsis CHMs, many bioactive herbal compounds belonging to the flavonoids, monoterpene glycosides, catechols, phthalides, ginsenosides, steroidal saponins, etc., have been identified as possessing antisepsis-related pharmacological activities and showing significant systemic exposure for exhibiting bioactivities after the administration of medicines. In the future, the elucidation of the material basis of antisepsis CHMs will require joint multidisciplinary efforts to provide an important basis for clarifying the pathogenesis of sepsis and developing novel antisepsis drugs.

## Figures and Tables

**Table 1:** Chemical compositions, pharmacological actions, bioactive compounds, and mechanisms of action of representative antisepsis Chinese herbal medicines.

Prescription	Component Herbs	Chemical Composition	Pharmacological Actions	Bioactive and Bioavailable Compounds	Potential Target Pathway	Potential DDI Target	References
XueBiJing injection	*Carthamus tinctorius* flower (Honghua in Chinese), *Paeonia lactiflora* root (Chishao), *Ligusticum chuanxiong* rhizome (Chuanxiong), *Angelica sinensis* root (Danggui), and *Salvia miltiorrhiza* root (Danshen)	Flavonoids, monoterpene glycosides, catechols, phthalides, organic acids, etc.	Exhibit anti-inflammatory, anticoagulant, endothelium-protective, immunoregulatory, antioxidant, and organ-protective activities; inhibit ox-LDL-induced apoptosis; improve microcirculation and myocardial ischemia/reperfusion injury	Hydroxysafflor yellow A	TLR4/NF-κB; NLRP3; Rac1/Akt; NF-κB/ICAM-1	—	[59,61,62,76]
				Paeoniflorin Oxypaeoniflorin Albiflorin	SIRT1; IRAK1-NF-κB; IκB; PI3K/Akt; TLR2; Sirt1/Foxo1	—	[33,57,60,77,78,79]
				Senkyunolide I	p-Erk1/2; Nrf2/HO-1; Caspase 3; MAPK; TLRs	As victim: UGT2B15	[29,33,64,80]
				Tanshinol	cAMP-PKA	As victim: OAT1/2	[29,58]
ShenFu injection	*Panax ginseng* steamed root (Hongshen) and processed *Aconitum carmichaelii* root (Fuzi)	Ginsenosides, aconitum alkaloids, organic acids, etc.	Regulate oxidative stress and inflammatory responses, inhibit HMGB1-mediated severe inflammatory response, restore endothelial integrity, attenuate the proinflammatory response, enhance innate immunity, preserve adaptive immunity, alleviate neuropathic pain	Ginsenosides Rb_1_, Rc, Rb_2_, Rf, Rd, Rg_1_, etc.	TLR4; PXR/NF-κB; TLRs/IRAK-1; TBK-1/IκB kinase ε/IRF-3; p38/ATF-2	As substrate: OATP1B3 (for Ginsenosides Rg_1_, Rf) As perpetrator: OATP1B1/1B3 (for Ginsenosides Rb_1_, Rc, Rb_2_, Rd)	[81,82,83,84,85,86,87,88,89,90,91,92,93]
				Benzoylmesaconine Fuziline Mesaconine Neoline Songorine	TLR4/NF-κB; Nrf2	As victim: P-gp (for benzoylmesaconine)	[94,95,96,97]
ShengMai formula	*Panax ginseng* root (Renshen), *Ophiopogon japonicus* root (Maidong), and *Schisandra chinensis* fruit (Wuweizi)	Ginsenosides, lignans, steroidal saponins, and homoisoflavanones	Exhibit anti-inflammatory or antioxidant, hepatoprotective activities	Ginsenosides Rb₁, Rb_2_, Rc, Rd, Re, Rg_1_, Rh_1_, Compound K, Rf, and Rg_2_	TLR4; PXR/NF-κB; TLRs/IRAK-1; TBK-1/IκB kinase ε/IRF-3; p38/ATF-2	As substrate: OATP1B1/1B3 (for Ginsenoside Rg_2_) OATP1B3 (for Ginsenosides Rg_1_, Rf, Re) As perpetrator: OATP1B1/1B3 (for Ginsenosides Rb_1_, Rc, Rb_2_, Rd) NTCP (for Rg1) CYP3A (for Rd)	[81,82,83,84,85,86,87,88,89,90,91,92,93,98,99,100,101]
				Ophiopogonin D Ophiopogonin D’ Ruscogenin	PPARα; NF-κB/IκBα; SIRT1; TLR4; TLR4/NF-κB/MyD88	As perpetrator: CYP3A4, 2C9, and 2E1 (for Ophiopogonin D) UGT1A6/1A8 (for Ophiopogonin D) UGT1A6/1A10 (for Ophiopogonin D’) NTCP (for Ophiopogonin D’) CYP3A (for Ophiopogonin D) As victim: OATP1B1/1B3 (for Ophiopogonin D)	[92,101,102,103,104,105,106]
				Schisandrol A Schisandrol B Schizandrin A Schizandrin B Deoxyschisandrin	iNOS; COX-2; PGE2; MAPK; TLR4/NF-κB/MyD88	As perpetrator: NTCP (for Schizandrin A)	[100,107,108,109,110]
Qingwen Baidu decoction	*Rehmannia glutinosa* root (Dihuang), *Rhinoceros unicornis* horn (Xijiao), *Coptidis chinensis* rhizome (Huanglian), *Gardenia jasminoides* fruit (Zhizi), *Platycodon grandiflorum* root (Jiegeng), *Scutellaria baicalensis* root (Huangqin), *Anemarrhena asphodeloides* rhizome (Zhimu), *Paeonia lactiflora* root (Chishao), *Scrophularia ningpoensis* root (Xuanshen), *Forsythia suspense* fruit (Lianqiao), *Lophatherum gracile* stem and leaf(Danzhuye), *Glycyrrhiza uralensis* root and rhizome (Gancao), *Paeonia suffruticosa* root cortex (Danpi), and *Gypsum Fibrosum* (Shigao)	Alkaloids, iridoids, flavonoids, etc.	Reduce LPS-induced intestinal damage; treat inflammation; alleviate LPS-induced acute kidney injury; alleviate liver injury in sepsis; exhibit anti-inflammatory, antioxidant, and cardioprotective effects	Berberine	TLRs; NF-κB; STAT3; Wnt/β-catenin; PI3K/Akt; MAPK/JNK/p38/ERK	As perpetrator: CYP3A4, CYP2D6 As victim: P-gp	[111,112,113,114,115,116,117]
				Geniposide Genipin	NF-κB; MAPK; PPARγ; AMPK; NLRP3; AKT-mTOR	—	[118,119,120,121,122,123]
				Baicalin	iNOS; COX-2; NF-κB; HMGB1	As perpetrator: CYP1A2/3A/2E1, OATP1B1, P-gp	[124,125,126,127,128]
				Wogonoside Wogonin	TLR4; NF-κB; Nrf2; NLRP3	As perpetrator: CYP1A2 (for Wogonin)	[129,130,131,132,133]
				Oroxylin A	JAK/STAT; IRF2BP2-NFAT1; NF-κB	As perpetrator: CYP1A2, OATP1B1, OAT1/3 and BCRP	[133,134,135,136,137,138,139,140]
				Verbascoside	iNOS	—	[141,142,143]
XuanBai Chengqi decoction	*Rheum palmatum* rhizome and root (Dahuang), *Gypsum Fibrosum* (Shigao), *Prunus armeniaca* seed (Kuxingren), and *Trichosanthes kirilowii* fruit (Gualou)	Anthraquinones, etc.	Attenuate LPS-induced microcirculatory disturbance	Emodin	TLR4/NF-κB/ICAM-1; JAK1/STAT3; MAPK; cAMP-PKA; NLRP3; PPARγ	As victim: CYP1A2, UGT1A8/1A10/12B7	[144,145,146,147,148,149,150,151]

## Data Availability

Not applicable.

## Abbreviations

ADE	Adverse drug event
ADR	Adverse drug reaction
ALuI	Acute lung injury
ALT	Alanine aminotransferase
AMP	Adenosine 5′-monophosphate
APACHE II	Acute Physiology and Chronic Health Evaluation II
ARDS	Acute respiratory distress syndrome
AST	Aspartate aminotransferase
BUN	Blood urea nitrogen
*C*_max_	Maximum plasma concentrations
COVID-19	Coronavirus disease 2019
CHM	Chinese herbal medicines
CLP	Cecal ligation and puncture
Cr	Creatinine
HLA-DR	Human leukocyte antigen-DR
ICU	Intensive care unit
LDH	Lactate dehydrogenase
IFN-γ	Interferon-γ
IL-6	Interleukin 6
LPS	Lipopolysaccharide
MAP	Mean arterial pressure
MLC	Myosin light chain
MLCK	Myosin light-chain kinase
MMP-2/9	Matrix metalloproteinase 2/9
MODS	Multiple organ dysfunction syndrome
OGD/R	Oxygen–glucose deprivation/reoxygenation
PEEP	Positive end-expiratory pressure
PSCC	Preventing Sepsis Campaign in China
QWBD	Qingwen Baidu decoction
ROCK	Rho-associated coil-forming protein kinase
SCCM	Society of Critical Care Medicine
SIRS	Systemic inflammatory response syndrome
20(*S*)-protopanaxadiol type	ppd-type
20(*S*)-protopanaxatriol type	ppt-type
SF	ShenFu injection
SM	ShengMai
SOCS1	Suppressor of cytokine signaling 1
SOFA	Sequential Organ Failure Assessment
SIRS	Systemic inflammatory response syndrome
*t*_1/2_	Half-life
TCM	Traditional Chinese medicine
TNF-α	Tumor necrosis factor-α
XBCQ	Xuanbai Chengqi decoction
XBJ	XueBiJing injection
